# Early proteomic signatures of Alzheimer’s disease in the retina and brain of 3xTg-AD mice

**DOI:** 10.3389/fcell.2026.1827348

**Published:** 2026-05-18

**Authors:** Artjola Puja, Rachel McNeel, Rong Xu, Siyan Zhu, David Hansman, Jianhai Du

**Affiliations:** 1 Department of Ophthalmology and Visual Sciences, West Virginia University, Morgantown, WV, United States; 2 Department of Biochemistry and Molecular Medicine, West Virginia University, Morgantown, WV, United States; 3 Department of Pharmaceutical and Pharmacological Sciences, West Virginia University, Morgantown, WV, United States

**Keywords:** alzheimer’s disease, brain, mitochondria, neurodegenerative disorders, proteomics, retina

## Abstract

**Introduction:**

Visual dysfunction and retinal structural changes can occur early in Alzheimer's disease (AD), but the molecular basis of these early alterations remains unclear.

**Methods:**

We performed quantitative proteomic profiling of the retina and brain from 4-week-old triple-transgenic AD (3xTg-AD) mice carrying human PS1M_146V_, APP_Swe_, and tau_P301L_ mutations, prior to detectable retinal morphological abnormalities.

**Results:**

Retinal morphology was normal in 4-week-old 3xTg-AD. Proteomic analysis identified 92 significantly altered proteins in the retina and 130 in the brain, with eight proteins overlapping between tissues. These overlapping proteins included three hemoglobin subunits and five proteins involved in protein homeostasis and vesicular transport. The retinal proteome was characterized by reduced vision-related proteins, altered small-molecule transporters, and decreased levels of proteins involved in mitochondrial energetics. In the brain, prominent changes were observed in mitochondrial proteins, including respiratory chain components and mitochondrial ribosomal subunits, as well as proteins linked to autophagy and synaptic vesicle pathways.

**Discussion:**

These findings identify early, common and tissue-specific proteomic changes in the retina and brain of 3xTg-AD mice prior to detectable retinal structure abnormalities. The data indicate early changes in proteins related to mitochondrial function and intracellular transport and support the use of retina as an accessible tissue for detecting preclinical AD pathology.

## Introduction

Alzheimer’s disease (AD) is a progressive neurodegenerative disorder and the leading cause of dementia worldwide (Perl, 2010; Rajan et al., 2021). Currently, there is no effective treatment to halt or reverse disease progression, and interventions started after clinical onset may contribute to the high failure of clinical trials (Dubois et al., 2014; Lane et al., 2018; Kim et al., 2022). These limitations underscore the need to identify early biomarkers and define disease mechanisms during the preclinical stage of AD. The classical neuropathological hallmarks of AD are extracellular amyloid-β (Aβ) plaques and intracellular neurofibrillary tangles composed of hyperphosphorylated tau protein (Ratan et al., 2023). Aβ peptides, predominantly Aβ40 and the more aggregation-prone Aβ42, are generated from amyloid precursor protein (APP) through sequential proteolytic cleavage by β- and γ-secretases. Mutations in presenilin-1 (PS1), a core component of the γ-secretase complex, alter APP processing and preferentially increase Aβ42 production, thereby accelerating Aβ accumulation, synaptic dysfunction, and cognitive decline (Suzuki et al., 1994; Selkoe, 2000; Annaert and De Strooper, 2002; Sun et al., 2017). However, AD is now recognized as a multifactorial disorder that extends beyond Aβ and tau pathology. Emerging evidence highlights early mitochondrial and metabolic dysfunction, impaired proteostasis, neuroinflammation, and vascular contributions as integral components of disease progression (Di Filippo et al., 2010; Gadhave et al., 2021; Tian et al., 2022; Ottoy et al., 2023; Kazemeini et al., 2024). Importantly, AD-associated pathology is not restricted to the brain but also affects other regions of the central nervous system (CNS), particularly the visual system (Bayer et al., 2002; Jiang et al., 2016; La Morgia et al., 2016).

Visual dysfunction is increasingly recognized as an early feature of AD, preceding the onset of cognitive symptoms (London et al., 2013; Cerquera-Jaramillo et al., 2018). The retina, an accessible extension of the CNS, provides a unique window to assess the brain pathology (Chow and Lang, 2001; London et al., 2013). Structural and functional retinal abnormalities have been reported in AD patients, including thinning of the retinal nerve fiber layer (RNFL) and ganglion cell layer (GCL), reduced contrast sensitivity, and altered pupillary responses (Kim and Kang, 2019; Kremen et al., 2019; Risacher et al., 2020). Importantly, some of these retinal alterations correlate with cortical Aβ burden and cognitive impairment (On behalf of the FACEHBI study group, 2020; Song et al., 2021), suggesting that retinal changes may mirror early neurodegenerative processes occurring in the brain. The retina is among the most metabolically active tissues in the body and is highly dependent on mitochondrial function and efficient nutrient transport to maintain visual processing (Wong-Riley, 2010; Country, 2017; Hanna et al., 2022). This high bioenergetic demand may render retinal tissue particularly sensitive to early metabolic and mitochondrial disturbances that characterize AD pathogenesis (Yaffe, 2004; Dumont et al., 2009; Yang et al., 2022; Zhang et al., 2025).

To date, studies investigating retinal involvement in AD have largely emphasized structural and functional assessments, such as optical coherence tomography and electroretinography (Blanks et al., 1996; Koronyo-Hamaoui et al., 2011; Kim and Kang, 2019; Asanad et al., 2021). However, early molecular alterations in the retina during AD progression remain poorly characterized. Furthermore, it remains unclear if the retina shows similar early biochemical alterations as the brain. To this end, we investigated early proteomic changes in the retina and brain using triple-transgenic AD (3xTg-AD) mouse model, harboring PS1_M146V_, APP_Swe_, and tau_P301L_ mutations (Oddo et al., 2003). In this model, detectable brain pathology typically emerges around 4–6 months of age, with intracellular Aβ accumulation beginning at 3–4 months and extracellular plaques appearing at 6–9 months, whereas 4-week-old mice do not yet show functional deficits (Belfiore et al., 2019; Javonillo et al., 2021). We therefore focused on 4-week-old 3xTg-AD mice to capture early, pre-symptomatic alterations. We assessed retinal morphology and performed comprehensive quantitative proteomic analyses on both retina and brain tissues to identify early proteomic signatures in AD.

## Methods

### Animals

Female 3xTg-AD on a B6; 129 genetic background (JAX #034830) and age-matched wild-type (WT) controls on a B6; 129 background (JAX #101045) were all purchased from the Jackson Laboratory. Mice were housed and aged in the West Virginia University vivarium under a 12-h light/12-h dark cycle and maintained on a standard chow diet (2018 Teklad Global 18% Protein, Inotiv Inc.) and water available *ad libitum*. All animal procedures were conducted in compliance with the National Institutes of Health guidelines and ARVO Statement for the Use of Animals in Ophthalmic and Vision Research. Experimental protocols were approved by the Institutional Animal Care and Use Committee (IACUC) of West Virginia University. Animals were genotyped by Transnetyx using polymerase chain reaction (PCR) analysis of genomic DNA isolated from tail samples.

### Hematoxylin and eosin staining and analysis

Mice were sacrificed at 4 weeks and 28 weeks of age, and their eyes were carefully enucleated and immediately fixed in 1 mL of Excalibur’s Alcoholic Z-Fix (Excalibur Pathology Inc., Norman, OK). Tissues were subsequently processed, paraffin-embedded, sectioned, and stained with hematoxylin and eosin (H&E) by Excalibur Pathology. H&E-stained retinal sections were imaged using a Nikon Eclipse Ti microscope equipped with a DS-Ri2 camera (Nikon Instruments, Melville, NY). Thickness of the outer segments (OS), inner segments (IS), outer nuclear layer (ONL), and inner nuclear layer (INL) was quantified using ImageJ at six predefined locations across the retina near the optic nerve head (ONH) on both the inferior and superior sides of the eye, as previously described (Grenell et al., 2019). Briefly, three positions were defined on each side of the ONH (−1, −2, −3 and +1, +2, +3), with −1 and +1 located 20 nuclei away from the ONH, −3 and +3 located 20 nuclei inward from the peripheral retina, and −2 and +2 defined as intermediate points between these locations. Each measurement was replicated ten nuclei away from the first measurement. For each eye, ten sections were analyzed and values were averaged to obtain the final thickness.

### Tissue collection and protein extraction

Mice at 4 weeks of age were euthanized, and the eyecups were immediately enucleated (N = 4 eyecups per group). Retinal dissections were performed in cold Hanks' Balanced Salt Solution (HBSS) to isolate the neural retina, as previously described (Zhu et al., 2018; Xu et al., 2020). Whole brains were excised and briefly rinsed in cold Phosphate Buffered Saline (PBS). All tissues were flash-frozen in liquid nitrogen and stored at −80 °C until further processing. For protein extraction, retina and brain samples were homogenized in radioimmunoprecipitation assay (RIPA) buffer supplemented with protease and phosphatase inhibitors (5 mg/mL). Homogenates were centrifuged at 12,000 rpm for 15 min at 4 °C, and the supernatants containing soluble proteins were collected. Protein concentrations were determined using the Pierce™ BCA Protein Assay Kit (see Table 1), and equal amounts of total protein were used for downstream digestion and mass spectrometry analysis.

**TABLE 1:** Key resources.

Mouse strain	Catalog	Company	Location
3xTg-AD	MMRRC_034830-JAX	The Jackson Laboratory	Bar Harbor, ME, United States of America
B6129SF1/J	IMSR_JAX:101043	The Jackson Laboratory	Bar Harbor, ME, United States of America
Mouse diet	Catalog	Company	Location
2018 Teklad Global 18% Protein Rodent Diet	2018	Inotiv Inc	West Lafayette, IN, United States of America
Reagents	Catalog	Company	Location
RIPA Lysis and Extraction Buffer	89901	Thermo Fisher Scientific	Rockford, IL United States of America
Pierce Protease and Phosphatase Inhibitor Mini Tablets	A32959	Thermo Fisher Scientific	Rockford, IL United States of America
Pierce™ BCA Protein Assay Kit	23225	Thermo Fisher Scientific	Rockford, IL United States of America
Excalibur’s Alcoholic Z-Fix	1024	Excalibur Pathology Inc	Norman, OK United States of America
Phosphate Buffer Saline	3013794	Thermo Fisher Scientific	St. Louis, MO United States of America
Hanks’ Balanced Salt Solution	2276814	Thermo Fisher Scientific	Waltham, MA United States of America

### Quantitative proteomics

Total proteins from each sample were reduced, alkylated, and purified by chloroform/methanol extraction prior to digestion with sequencing grade modified porcine trypsin (Promega). Tryptic peptides were then separated by reverse phase XSelect CSH C18 2.5 um resin (Waters) on an in-line 150 × 0.075 mm column using an UltiMate 3,000 RSLCnano system (Thermo). Peptides were eluted using a 60 min gradient from 98:2 to 65:35 buffer A:B ratio (Buffer A contains 0.1% formic acid, 0.5% acetonitrile. Buffer B contains 0.1% formic acid, 99.9% acetonitrile). Eluted peptides were ionized by electrospray (2.2 kV) followed by mass spectrometric analysis on an Orbitrap Exploris 480 mass spectrometer (Thermo). To assemble a chromatogram library, six gas-phase fractions were acquired on the Orbitrap Exploris with 4 m/z DIA spectra (4 m/z precursor isolation windows at 30,000 resolution, normalized AGC target 100%, maximum inject time 66 m) using a staggered window pattern from narrow mass ranges using optimized window placements. Precursor spectra were acquired after each DIA duty cycle, spanning the m/z range of the gas-phase fraction (i.e. 496-602 m/z, 60,000 resolution, normalized AGC target 100%, maximum injection time 50 m). For wide-window acquisitions, the Orbitrap Exploris was configured to acquire a precursor scan (385–1015 m/z, 60,000 resolution, normalized AGC target 100%, maximum injection time 50 m) followed by 50 × 12 m/z DIA spectra (12 m/z precursor isolation windows at 15,000 resolution, normalized AGC target 100%, maximum injection time 33 m) using a staggered window pattern with optimized window placements. Precursor spectra were acquired after each DIA duty cycle.

#### Proteomics data processing

Following data acquisition, data were searched using an empirically corrected library and a quantitative analysis was performed to obtain a comprehensive proteomic profile. Proteins were identified and quantified using EncyclopeDIA (Searle et al., 2018) and visualized with Scaffold DIA using 1% false discovery thresholds at both the protein and peptide level. Protein intensity values were normalized across samples using an in-house ProteiNorm pipeline (Graw et al., 2020), a tool for systematic evaluation of normalization methods, imputation of missing values, and comparisons of multiple differential abundance methods. Normalization methods evaluated included log2 normalization (Log2), median normalization (Median), mean normalization (Mean), variance stabilizing normalization (VSN) (Huber et al., 2002), quantile normalization (Quantile) (Bolstad et al., 2003), cyclic loess normalization (Cyclic Loess) (Ritchie et al., 2015), global robust linear regression normalization (RLR), and global intensity normalization (Global Intensity) (Chawade et al., 2014). The individual performance of each method was evaluated by comparing of the following metrices: total intensity, pooled intragroup coefficient of variation (PCV), pooled intragroup median absolute deviation (PMAD), pooled intragroup estimate of variance (PEV), intragroup correlation, sample correlation heatmap (Pearson), and log2-ratio distributions. The normalized data were used to perform statistical analysis using linear models for microarray data (limma) with empirical Bayes (eBayes) smoothing to the standard errors (Ritchie et al., 2015). Proteins with an FDR adjusted p-value <0.05 and a fold change (FC) > 2 were considered significant. Data are available via PRoteomic IDEntifications Database (PRIDE) with identifier PXD069995.

#### Bioinformatics analysis

Proteomics quantification results were further processed using bioinformatic platforms. Principal Component Analysis (PCA) and Variable Importance in Projection (VIP) scoring were performed using MetaboAnalyst (https://www.metaboanalyst.ca). Functional enrichment analysis of biological processes and cellular components for significantly changed proteins between 3xTg-AD and WT tissues, were conducted using the DAVID Bioinformatics Resources (https://davidbioinformatics.nih.gov). Protein-protein interaction network analysis was carried out using Metascape (https://metascape.org). Heatmaps and quantitative data visualizations were generated using GraphPad Prism (v9.5.1, GraphPad Software Inc.). To identify AD-associated proteins enriched in the human brain proteomic dataset, we queried the Agora AD Knowledge Portal (https://agora.adknowledgeportal.org). Identified associations were further validated through manual curation of published literature using PubMed, with combinations of relevant keywords (e.g., “[protein name] AND [Alzheimer’s disease OR neurodegenerative disease]”).

### Statistics

The statistical analyses of retinal thickness are presented as the mean ± standard deviation (SD). Unpaired two-tailed t tests were performed in GraphPad Prism v9.4.1 to determine significance. Values p<0.05 were considered significant.

## Results

### Age-dependent retinal thinning in 3xTg-AD mice

**FIGURE 1:**
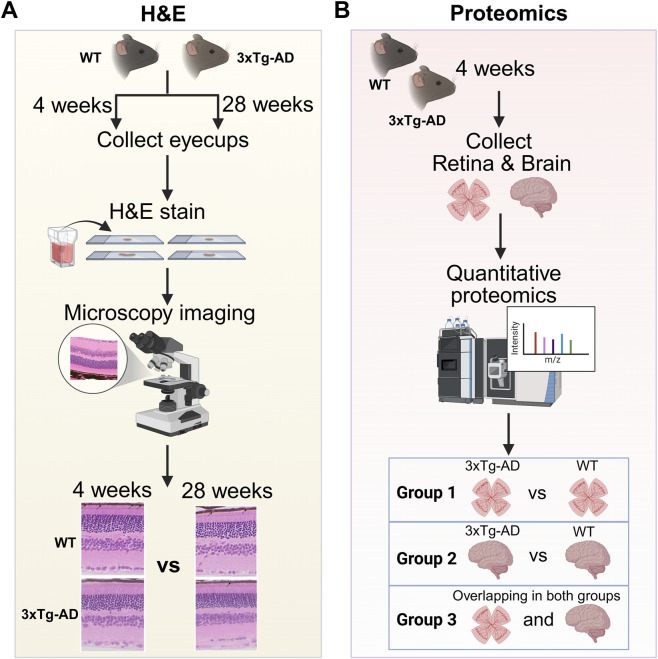
Experimental workflow. **(A)** Retinal morphology was assessed by Hematoxylin and Eosin (H&E) staining of eyecup sections from 3xTg-AD mice and WT controls at 4- and 28-weeks of age. **(B)** Quantitative proteomics was performed on retina and brain tissues from 4-week-old 3xTg-AD mice and WT controls.

**FIGURE 2:**
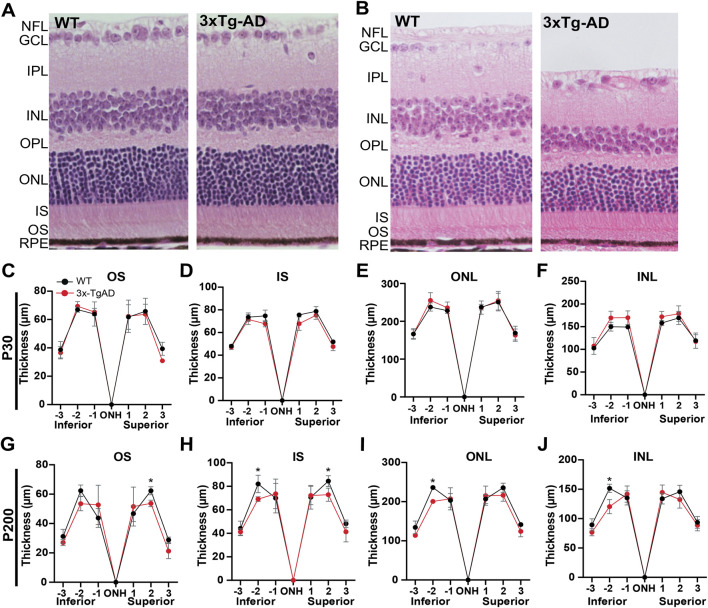
Age-associated structural alterations in the retina of 3xTg-AD mice. Representative H&E-stained retinal sections from WT and 3xTg-AD mice at **(A)** 4-week and **(B)** 28-week of age. **(C–F)** Quantification of retinal layer thickness at 4-weeks at the indicated positions from the optic nerve head (ONH). **(G–J)** Quantitative assessment of the thickness of retinal layers at 28-week. Asterisks indicate significant differences determined through *t* tests between the WT and 3xTg-AD groups. Data is presented as mean ± SD. RPE, retinal pigment epithelium; OS, outer segment; IS, inner segment; ONL, outer nuclear layer; INL, inner nuclear layer; IPL, inner plexiform layer; GCL, ganglion cell layer; NFL, Nerve fiber layer. N = 4 eyecups/group.

To examine retinal morphological changes, we quantified the thickness of individual retinal layers in H&E-stained sections at 4-week-old and 28-week-old mice. At 4-week, retinal structure in 3xTg-AD mice was comparable to that of WT controls, with no differences across retinal layers (Figures 1A, 2A,C–F). At 28 weeks, selective reductions in retinal layer thickness were observed in 3xTg-AD mice at specific locations, affecting OS, IS, ONL, and INL (Figures 2G–J). This progressive decline in retinal thickness with age in 3xTg-AD mice is consistent with reports of retinal layer thinning in AD patients (Pillai et al., 2016; Cunha et al., 2017; Kim and Kang, 2019).

#### Early retinal proteomic alterations in 3xTg-AD mice

**FIGURE 3:**
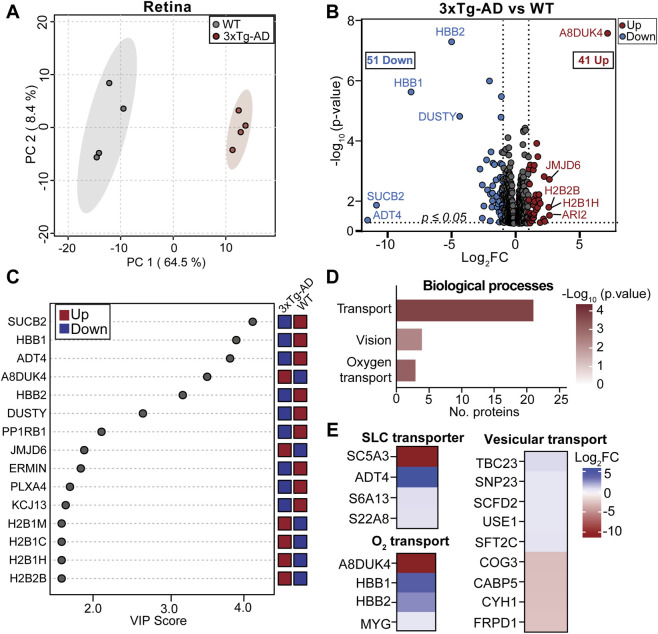
Early retinal proteomic changes in 3xTg-AD mice. **(A)** Two-dimensional principal component analysis (PCA) plot shows clear separation between 3xTg-AD and WT retina proteome. **(B)** Volcano plot of differentially expressed proteins in the retina of 3xTg-AD compared to WT. Top five up- and downregulated are labeled (|log_2_FC| ≥ 1, p ≤ 0.05; red = upregulated, blue = downregulated in 3xTg-AD). **(C)** Variable Importance in Projection (VIP) plot highlighting the top 15 proteins that separate 3xTg-AD from WT retina. Tiles on the right indicate up- (red) and downregulated (blue). **(D)** Biological pathways enriched in 3xTg-AD identified by Gene ontology (GO) analysis. **(E)** Heatmaps of transport-related processes with representative proteins enriched in 3xTG-AD versus WT. SLC, solute carrier transporter. N = 4.

To investigate early proteomic alterations preceding AD pathology, we performed quantitative proteomic analysis of retinas from 3xTg-AD and WT mice at 4 weeks of age (Figure 1B). Principal component analysis (PCA) showed a clear separation between two groups, indicating early global proteomic change in the retina from 3xTg-AD mice (Figure 3A). A total of 92 proteins were significantly altered in 3xTg-AD retinas, including 51 downregulated and 41 upregulated proteins (Figure 3B; Supplementary Table S1). Variable Importance in Projection (VIP) analysis identified top 15 proteins driving PCA group separation (Figure 3C) with prominent changes of mitochondrial proteins involved in energy metabolism, such as succinate-CoA ligase subunit β (SUCB2) and ADP/ATP translocase 4 (ADT4), as well as hemoglobin-related proteins (HBB1, HBB2, and A8DUK4).

Specifically, proteins associated with retinal structure and neuronal connectivity, including ermin (ERMIN) and plexin-A4 (PLXA4), whereas multiple histone H2B variants (H2B1M, H2B1C, H2B1H, H2B2B) and the histone demethylase JMJD6 were increased (Figure 3C). Gene ontology (GO) analysis showed enrichment of pathways related to transport, in particular oxygen transport, and vision (Figures 3D,E; Supplementary Table S2). Several solute carrier (SLC) transporters were differentially expressed in 3xTg-AD retina. SC5A3 was significantly upregulated, whereas ADT4, S6A13, and S22A8 were downregulated (Figure 3E). Notably, ADT4, a mitochondrial ADP/ATP translocase, was reduced by more than 11-fold, suggesting impaired mitochondrial energy output. In addition, proteins involved in vesicular transport were broadly altered. Components associated with endoplasmic reticulum (ER)-Golgi trafficking and synaptic vesicle dynamics, including TBC23, SNP23, SCFD2, USE1, and SFT2C, were decreased. In contrast, expression of proteins involved in Golgi organization, calcium-dependent synaptic signaling, and vesicle recycling such as COG3, CABP5, CYH1, and FRPD1, was increased. Strikingly, vision-related proteins essential for photoreceptor maintenance and retinal signaling, including RGR, RDH5, RPE65, IMPG2, and KCNJ13, were uniformly downregulated (Supplementary Figure S1). Together, these findings demonstrate that proteins associated with chromatin, mitochondrial metabolism, intracellular transport, and vision are altered early in the retina of 3xTg-AD mice.

##### Coupled mitochondrial and nuclear protein alterations in the AD retina

**FIGURE 4:**
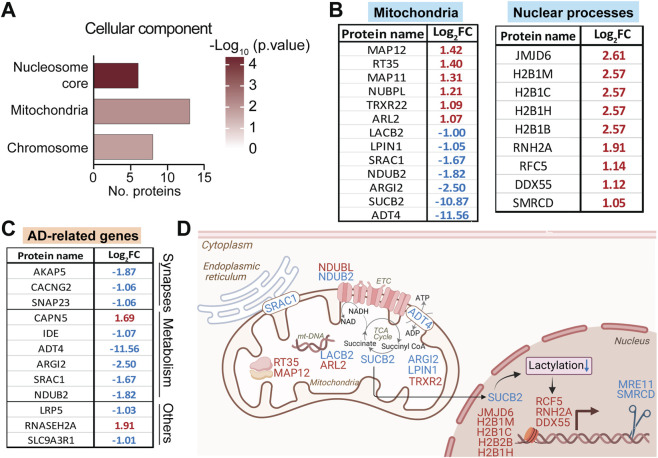
Differential mitochondrial and nuclear proteome changes in 3xTg-AD retina. **(A)** GO enrichment for cellular components altered in 3xTg-AD retina. **(B)** Differentially expressed mitochondria- and nucleus-related proteins in the retina (red = upregulated, blue = downregulated in 3xTg-AD vs. WT). **(C)** Proteins differentially expressed in 3xTg-AD retina that are linked to AD pathology, identified using Agora AD Knowledge Portal and manual literature search. **(D)** Proposed working model illustrating how mitochondrial proteome changes may signal to the nucleus and reprogram nuclear gene expression in the retina. See Supplementary Table S1 for detailed protein annotations and fold changes. AD, Alzheimer’s disease; mt-DNA, mitochondrial DNA; ETC., electron transport chain; TCA cycle, tricarboxylic acid cycle.

Building on the pathway-level dysregulation identified in early AD retinas, we next examined the subcellular localization of differentially expressed proteins. Cellular component enrichment analysis demonstrated that significantly altered proteins in 3xTg-AD retinas were predominantly localized to the nucleosome core, mitochondria, and chromosome (Figure 4A; Supplementary Table S2). Multiple mitochondrial proteins involved in metabolism and energy production, including LACB2, LPIN1, SRAC1, NDUB2, ARGI1, SUCB2, and ADT4, were downregulated in the AD retina (Figure 4B). In contrast, proteins associated with nuclear processes were upregulated, including histone variants and factors involved in DNA replication (RFC5), RNA processing (RNH2A, DDX55), and chromatin remodeling (SMRCD).

To determine whether the significantly altered retinal proteins identified in 3xTg-AD mice have known associations with AD in humans, we queried the Agora AD Knowledge Portal to identify proteins linked to AD pathogenesis and to explore potentially shared pathways. We found that twelve of the significantly altered retinal proteins involved in synaptic function and metabolism have prior evidence linked to AD pathogenesis (Figure 4C). In particular, several mitochondrial proteins which are changed in the 3xTG-AD retina (e.g., ADT4, SRAC1, ARGI2) were also altered in AD patients. The reduction of multiple mitochondrial proteins suggests that mitochondrial dysfunction contributes to early retinal stress in AD (Figure 4D). In particular, decreased expression of SUCB2, the β-subunit of mitochondrial succinyl-CoA ligase, indicates disruption of TCA cycle-linked metabolism and of succinyl-CoA availability, a key donor for lysine succinylation and lactylation (Weinert et al., 2013; Hu et al., 2023; Liu et al., 2025). This retrograde mitochondria-to-nucleus signaling is known to influence nuclear gene expression (Hu et al., 2023; Liu et al., 2025; Tsusaka et al., 2025). Together, these findings indicate that mitochondrial dysfunction and associated nuclear responses represent an early molecular feature of retinal deficit in AD.

#### Early proteomic alterations in 3xTg-AD brain

**FIGURE 5:**
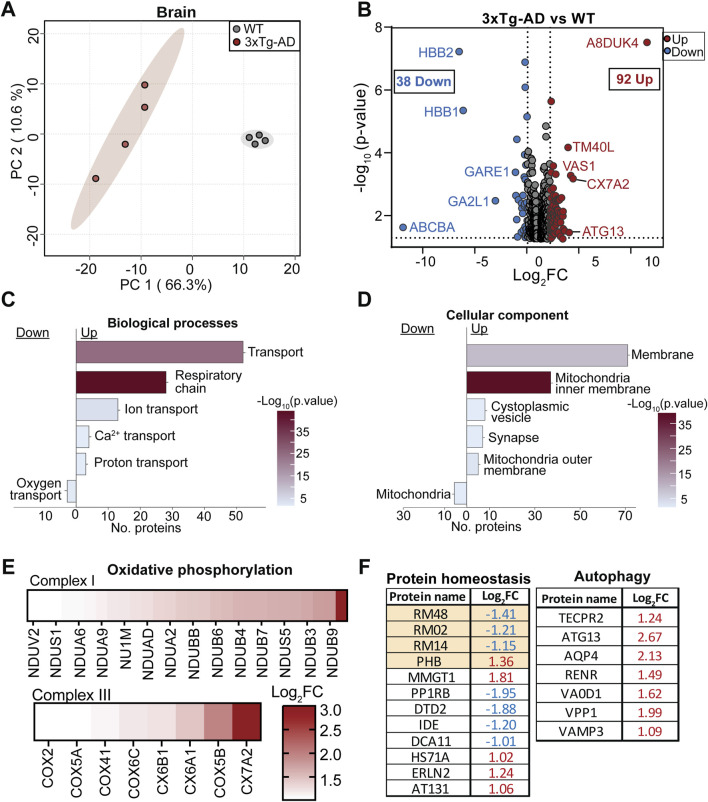
Early proteomic changes in the 3xTG-AD mouse brain. **(A)** Two-dimensional PCA plot shows clear separation between 3xTg-AD and WT brain proteome. **(B)** Volcano plot of differentially expressed proteins in the brain of 3xTg-AD compared to WT. Top five up- and downregulated are shown (|log2FC| ≥ 1, p ≤ 0.05; red = upregulated, blue = downregulated in 3xTg-AD). **(C)** GO enrichment analysis for biological processes and **(D)** cellular components altered in 3xTg-AD brain. **(E)** Enrichment of oxidative phosphorylation-related proteins in 3xTg-AD brain. **(F)** Differentially expressed 3xTg-AD brain proteins linked to protein homeostasis and autophagy, identified by PubMed literature search. Mitochondrial proteins are highlighted in orange. N = 4.

Multivariate PCA demonstrated a clear separation between 3xTg-AD and WT brain proteomes, indicating substantial global differences in protein expression (Figure 5A). Differential expression analysis identified 130 significantly altered proteins, including 38 downregulated and 92 upregulated in the AD brain (Figure 5B; Supplementary Table S3). Similar to the retina, hemoglobin subunits (HBB1, HBB2, and A8DUK4) were among the most prominently altered proteins. GO analysis of biological processes showed significant enrichment of pathways related to molecular transport and the mitochondrial respiratory chain in the 3xTg-AD brain (Figure 5C; Supplementary Table S4). Consistent with this, cellular component analysis indicated that differentially expressed proteins were primarily localized to mitochondria, membrane, cytoplasmic vesicles, and synaptic compartments (Figure 5D; Supplementary Table S4). Assessment of mitochondrial pathways showed broad changes in proteins involved in oxidative phosphorylation and electron transport chains, with strong enrichment of subunits from Complex I and Complex III (Figure 5E). These included multiple NADH dehydrogenase subunits, such as NDUS1, NDUS5, NDUA6, as well as cytochrome c oxidase–associated proteins, including COX5A, CX6A1, and CX7A2.

In addition to respiratory chain alterations, proteins involved in mitochondrial protein homeostasis and autophagy were also differentially expressed (Figure 5F). These included downregulation of ribosomal and mitochondrial-associated proteins such as large ribosomal subunit protein mL48 (RM48), uL2m (RM02), uL14 m (RM14), and prohibitin 1 (PHB), as well as upregulation of autophagy-related proteins including ATG13 and TECPR2. Protein-protein interaction network analysis further highlighted clustering of altered proteins within pathways related to oxidative phosphorylation, mitophagy, and synaptic vesicle maturation (Supplementary Figure S2). To assess the relevance of these proteomic changes to AD, we examined whether altered brain proteins have prior evidence of involvement in AD. Several proteins with established links to Aβ clearance, tau regulation, and neuroinflammation were altered in 3xTg-AD brain proteome, including downregulation of the Aβ-degrading enzyme IDE and the tau deacetylase HDAC6, and upregulation of the outer mitochondrial membrane proteins VDAC1 and VDAC2, the NF-κB kinase IKKβ, and the autophagy initiator ATG13 (see Table 2). Taken together, the proteomic alterations in the 3xTg-AD brain are dominated by changes in mitochondrial proteins including proteins with established relevance to AD pathogenesis.

**TABLE 2:** AD-relevant proteins altered in the brain proteome of 3xTg-AD mice at 4-weeks of age**.** Brain proteomic analysis of 3xTg-AD mice identified several proteins with established relevance to AD pathogenesis that were significantly altered at this early stage, prior to detectable amyloid or tau pathology. |log_2_FC| ≥ 1; p ≤ 0.05. Mitochondria outer membrane (MOM).

Protein ID	Full name	Log_2_FC	Localization	References
IDE	Insulin-degrading enzyme	−1.20	Cytoplasm	Hubin et al. (2016), Rowland et al. (2023)
TM40 L	Mitochondrial import receptor subunit TOM40B	2.58	MOM	Periasamy et al. (2022), Chen et al. (2023)
VDAC1	Voltage-dependent anion-selective channel protein 1	1.12	MOM	Yoo et al. (2001), Shoshan-Barmatz et al. (2018), Yang et al. (2024)
VDAC2	Voltage-dependent anion-selective channel protein 2	1.05	MOM	Yoo et al. (2001), Atlante et al. (2022)
HDAC6	Histone deacetylase 6	−1.21	Cytoplasm, Nucleus	Trzeciakiewicz et al. (2020)
IKKβ	Inhibitor of nuclear factor kappa-B kinase subunit beta	1.93	Cytoplasm	Schnöder et al. (2023)
ATG13	Autophagy-related protein 13	2.67	Cytoplasm	Uddin et al. (2018), Zhang et al. (2021)

### Comparison of early proteomic alterations in retina and brain in 3xTg-AD mice

**FIGURE 6:**
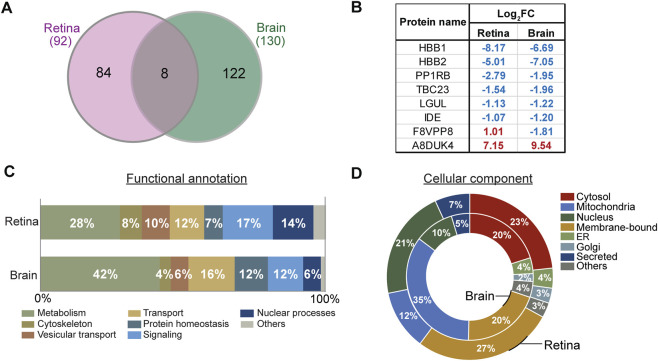
Comparison of differentially expressed proteins in retina and brain. **(A)** Total significantly changed and overlapping proteins in retina and brain. **(B)** Overlapping proteins between retina and brain. **(C)** Bar plots represent the percentage distribution of significantly changed proteins based on functional annotation and **(D)** cellular components in retina (outer plot) and brain (inner plot). ER, Endoplasmic Reticulum; Membrane-bound, includes proteins in cellular and intracellular membranes.

To investigate how early proteomic changes were shared between the retina and brain, we compared proteins significantly altered in each tissue of 3xTg-AD mice relative to WT controls. Among the 92 proteins altered in the retina and the 130 in the brain, eight proteins were significantly changed in both tissues (Figure 6A). These shared proteins included three hemoglobin-related proteins (HBB1, HBB2, and A8DUK4), and several proteins involved in metabolic regulation and protein turnover, including insulin-degrading enzyme (IDE), lactoylglutathione lyase (LGUL), E3 ubiquitin-protein ligase (PP1RB), TBC1 domain family member 23 (TBC23), and zinc finger CCCH-type containing protein 7B (F8VPP8). Except for A8DUK4 and F8VPP8, all overlapping proteins were reduced in both tissues (Figure 6B).

Pathway and cellular localization analyses further revealed common changes across the two tissues. GO analysis showed that metabolism, transport, and signaling were among the most highly enriched biological processes in both retina and brain (Figure 6C). Analysis of subcellular localization demonstrated that significantly altered proteins in both tissues were similarly distributed in cytosolic and membrane-bound compartments, while tissue-specific differences were observed in mitochondrial and nuclear fractions: mitochondrial proteins were more prominently altered in the brain (35% vs. 12% in retina), and nuclear proteins were more enriched in the retina (21% vs. 10% in brain), suggesting compartment-specific vulnerabilities in each tissue (Figure 6D). Together, these comparative analyses indicate that the retina and brain share early AD-associated proteomic alterations while also having tissue-specific changes in the initial stages of disease.

## Discussion

In this study, we have identified early proteomic signatures in the retina and brain of 3xTg-AD mice. Although only eight proteins overlapped between the two tissues, both showed similar alterations in mitochondrial metabolism, oxygen, and intracellular transport. In addition to common changes, the retinal proteome showed specific alterations in vision-related, transporter, and chromatin-associated proteins, whereas the brain proteome featured changes in mitochondrial respiratory chain components and protein homeostasis. Many of these dysregulated pathways are recognized as progressive drivers or early features of AD in patients (Decker et al., 2010; Di Filippo et al., 2010; Kundra et al., 2017; Kazemeini et al., 2024; Koronyo et al., 2023). Our findings further support the retina as a sensitive “window to the brain” for detecting preclinical AD pathology.

Among the shared alterations, β-globin subunits HBB1, HBB2, and A8DUK4 stand out as consistently dysregulated in both tissues. While hemoglobin is classically associated with erythrocytes, neurons and glial cells also express hemoglobin to facilitate oxygen uptake under hypoxic stress (Biagioli et al., 2009; Schelshorn et al., 2009). Altered hemoglobin expression and localization have been reported in AD brain, including enrichment near Aβ plaques and cerebral amyloid angiopathy (Wu et al., 2004; Ashraf et al., 2020), as well as paradoxical depletion in neurons containing neurofibrillary tangles (Ferrer et al., 2011; Altinoz et al., 2019). Beyond oxygen transport, neuronal hemoglobin has been implicated in mitochondrial function, mitochondria-to-nucleus signaling, epigenetic regulation, and autophagy (Ferrer et al., 2011; Codrich et al., 2017; Altinoz et al., 2019). In addition, hemoglobin can localize to the inner mitochondrial membrane (Shephard et al., 2014), interact with ATP synthase (Brown et al., 2016), enhance mitochondrial respiration, and improve neuronal oxygen utilization in dopaminergic neurons (Biagioli et al., 2009; Schelshorn et al., 2009). In contrast to the brain, the role of hemoglobin in the neural retina remains unclear. Human retinal pigment epithelial (RPE) cells can synthesize and secrete hemoglobin (Tezel et al., 2009). In addition, hemoglobin expression in retinal macroglia and ganglion cells increases under hypoxic conditions to support cell survival (Tezel et al., 2010). Given the high metabolic demand and relatively low physiological oxygen availability in the retina (Hurley, 2021), locally expressed hemoglobin may help support the mitochondrial function. Thus, altered expression of hemoglobin subunits in both retina and brain at this early stage of AD may reflect a compensatory adaptation to altered mitochondrial function. However, how neuronal hemoglobins contribute to AD pathogenesis requires further investigation.

The detection of multiple proteins with established relevance to AD pathology supports the sensitivity of our approach and confirms that the 3xTg-AD model recapitulates early molecular features of the disease (Table 2). Among them, IDE, HDAC6, VDAC1/2, and ATG13 were significantly changed in 3xTg-AD brain proteome. IDE is the primary cytosolic protease responsible for Aβ degradation; its downregulation reduces Aβ clearance and is thought to contribute to amyloid accumulation prior to plaque formation. Reduced IDE expression has been reported in AD brain, particularly in the hippocampus of APOE-ε4 carriers (Cook et al., 2003; Hubin et al., 2016; Rowland et al., 2023). HDAC6, a cytosolic deacetylase, is linked to tau modification and AD pathology. Both genetic loss and pharmacological inhibition of HDAC6 are associated with tau aggregation and altered autophagic clearance in AD models (Trzeciakiewicz et al., 2020; 2020; Qureshi and Chinnathambi, 2022). VDAC1 and VDAC2 are outer mitochondrial membrane channels known to interact with Aβ. VDAC1, the predominant isoform, is increased in AD brain and APP-transgenic models, where its abnormal interaction with Aβ and phosphorylated tau has been linked to impaired metabolite exchange, mitochondrial dysfunction, and apoptosis (Shoshan-Barmatz et al., 2018). VDAC2 is expressed at lower levels in neurons and plays a modest, region-dependent role in AD than VDAC1 (Yoo et al., 2001; Atlante et al., 2022). ATG13 is a core component of the autophagy initiation complex. Accumulating evidence has shown that impaired autophagy contributes to AD pathogenesis (Yu et al., 2005; Uddin et al., 2018; Zhang et al., 2021). However, whether changes in ATG13 reflect a compensatory response or an early maladaptive shift remains unclear.

Mitochondrial dysfunction is a key contributor in AD pathogenesis, with extensive evidence of impaired oxidative metabolism, reduced ATP production, altered tricarboxylic acid (TCA) cycle, and respiratory chain activity (Onyango et al., 2016; Buchman et al., 2019; Wang et al., 2020; 2024). Key metabolic enzymes SUCB2 and ADT4 were reduced more than 10- and 11-fold, respectively, in the 3xTg-AD retina (Figure 4B). SUCB2, encoded by *SUCLG2*, catalyzes the reversible conversion of succinyl-CoA to succinate coupled with GTP synthesis (Phillips et al., 2009). This reaction is particularly important in the retina, where GTP is required for cGMP production during phototransduction (Du et al., 2016). Beyond its metabolic role, SUCB2 is important for protein succinylation and lactylation, linking metabolism to post-translational modifications (Figure 4D) (Zhang et al., 2023; Liu et al., 2025). Altered succinylation of mitochondrial proteins, APP, and tau has been reported in human AD brains and mouse models (Pan et al., 2022; Yang et al., 2022; Zhang et al., 2025). Further studies will be required to examine if these modifications are altered in the retinal proteome at this stage. Similarly, ADT4 (*SLC25A31*), a mitochondrial ADP/ATP translocase responsible for exchanging matrix ATP with cytosolic ADP across the inner mitochondrial membrane, is massively reduced. Such impaired ADP/ATP exchange can compromise oxidative phosphorylation, alter mitochondrial membrane potential, and increase susceptibility to cellular stress (Clémençon et al., 2013; Gutiérrez-Aguilar and Baines, 2013; Jia and Du, 2021). The substantial reduction of SUCB2 and ADT4 in early AD retina could limit the availability of GTP and ATP, compromising mitochondrial metabolism and potentially affecting phototransduction and metabolite-driven signaling. It warrants future investigation whether these metabolic defects contribute to the downregulation of retinal proteins involved in the visual cycle, ion transport, and photoreceptor maintenance before visual impairment in 3xTg-AD mice and AD patients (Kusne et al., 2017; Kim and Kang, 2019). These findings further support that mitochondrial dysfunction emerges early in the retina in AD pathogenesis.

This study has several limitations. First, we examined a single mouse model at one early time point, which provides a snapshot of early proteomic changes rather than a longitudinal trajectory. Future studies tracking these alterations at multiple time points will be important for understanding how the proteomic alterations evolve with AD progression. Second, the 3xTg-AD model expresses human APP_Swe_ and tau_P301L_ under a Thy1-based neuronal promoter, which drives early neuronal overexpression of these transgenes at non-physiological levels (Caroni, 1997; Oddo et al., 2003). Thus, some retinal and brain proteomic alterations may reflect transgene-driven neuronal responses rather than fully recapitulating the molecular changes of sporadic AD. Because Thy1 activity in the retina is enriched in inner retinal neurons, the whole-retina proteome likely underrepresents cell types with little or no transgene expression (Chiu et al., 2012; Salobrar-García et al., 2020). Third, whole-brain analysis averages proteomic signals across multiple brain regions, which may obscure region-specific vulnerability. Future studies using cell type- and region-resolved proteomics will be needed to provide greater spatial resolution of these changes. Finally, the identified candidate proteins and pathways require further biochemical and functional validation to establish their significance in AD diagnosis and pathogenesis. Despite these limitations, our work establishes that the retina and brain share overlapping yet distinct early proteomic changes, underscoring the importance of multi-tissue omics approaches for capturing the early molecular landscape of AD.

## Data Availability

The datasets presented in this study can be found in online repositories. The names of the repository/repositories and accession number(s) can be found in the article/Supplementary Material.
