# Supplementary material for: Early proteomic signatures of Alzheimer’s disease in the retina and brain of 3xTg-AD mice
Source: Front Cell Dev Biol. 2026 May 18;14:1827348. doi: 10.3389/fcell.2026.1827348 (PMC13223019; doi:10.3389/fcell.2026.1827348)
Supplement: Supplementary file 2 [file DataSheet1.docx]

**Supplementary Information**

**Early proteomic signatures of Alzheimer Disease in the retina and brain of 3xTg-AD mice**

Artjola Puja^1,2†^, Rachel McNeel^1,2†^, Rong Xu^1,2^, Siyan Zhu^1,3^, David Hansman^1,2^, Jianhai Du^1,2^*

^1^Department of Ophthalmology and Visual Sciences, West Virginia University, Morgantown, WV 26505, USA
^2^Department of Biochemistry and Molecular Medicine, West Virginia University, Morgantown, WV 26505, USA
^3^Department of Pharmaceutical and Pharmacological Sciences, West Virginia University, Morgantown, WV 26506

This PDF file includes:

Supplementary Figures

**
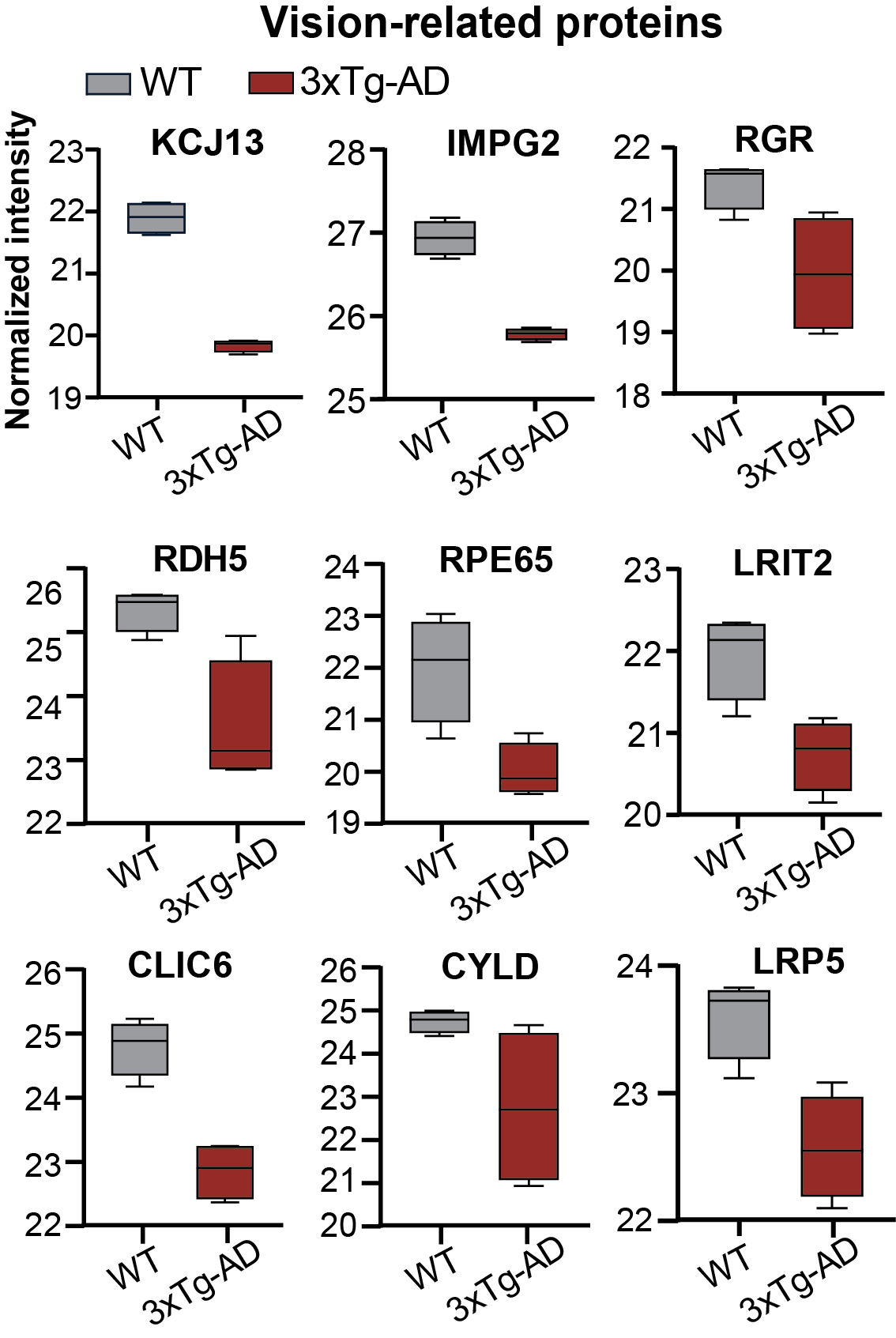
**

**Figure S1.** Bar charts of significantly changed vision-related proteins in the retina of 3xTg-AD compared to WT controls. N=4

**
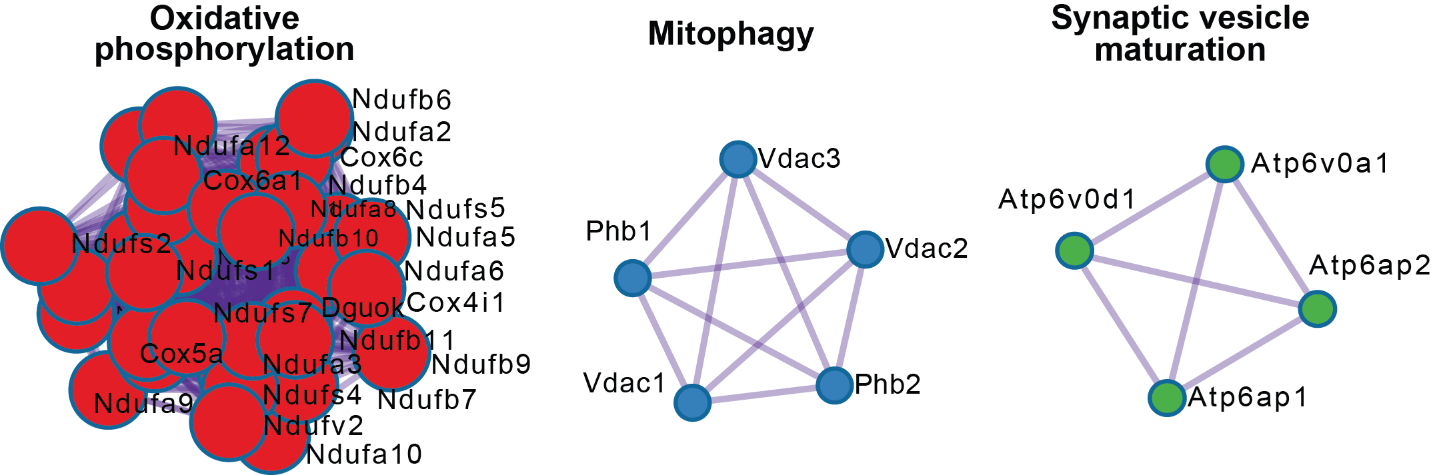
**

**Figure S2.** Clustering of the protein–protein interaction network of differentially expressed proteins in the brain tissues of 3xTg-AD mice. Analysis was performed using Metascape. N=4
